# Supplementary material for: Learning curve and surgical outcome of robotic assisted colorectal surgery with ERAS program
Source: Sci Rep. 2022 Nov 29;12:20566. doi: 10.1038/s41598-022-24665-w (PMC9709162; doi:10.1038/s41598-022-24665-w)
Supplement: Supplementary file 1 — Supplementary Information 1. [file 41598_2022_24665_MOESM1_ESM.zip › LSC Raw data-Ñ[▒K/076/P_ñw╝╨░O▒Kñσ.pdf]

臺中榮民總醫院 臺中市臺灣大道四段1650號 病理部活體組織切片報告 臨床醫學實驗及病理診斷中心 檢驗(查)報告

[redacted]

Pathologic diagnosis:
Colon, transverse to sigmoid, laparoscopic anterior resection --- a. Adenocarcinoma, moderately to poorly differentiated, with extensive necrosis. Tumor invaded to mesocolic soft tissue. The bilateral cut ends and mesocolic lymph nodes (0/47) are free of tumor. b. Ectopic spleen in omentum.

已電子簽章

Ancillary study for diagnosis:
1. IHS stains for SATB2 (+), synaptophysin (-), and chromogranin (-) done for section G; IHC stain for p40 (-) done for section F.
2. IHC stains for MLH1, PMS2 and MSH6 are done on section G.
- MLH1 and PMS2 show loss of nuclear expression.
- MSH6 shows preserved nuclear expression.

敬告：本單僅供醫師參考使用，不具任何申請效力！！
請病患妥為保管，若不慎遺失需自負個人資料外洩責任！

Prognostic and predictive factor:
1. Tumor size: 8x6 cm.
2. Depth of invasion: Mesocolic soft tissue.
3. Histologic grade: G2: Moderately differentiated to G3: Poorly differentiated.
4. Angiolymphatic invasion: Not identified.
5. Perineural invasion: Not identified.
6. Tumor deposits (discontinuous extramural extension): Not identified.
7. Macroscopic tumor perforation: Not identified.
8. Surgical Margins:
a) Proximal Margin: Uninvolved by invasive carcinoma, distance of tumor from margin: 3.5 cm.
b) Distal Margin: Uninvolved by invasive carcinoma, distance of tumor from margin: 21.5 cm.
c) Radial or Mesenteric Margin: Uninvolved by invasive carcinoma, distance of tumor from margin: 6 cm.
9. Lymph node status: Uninvolved (0/47).
10. Extranodal involvement: Not applicable.
11. Tumor regression grading S/P CCRT: No known presurgical therapy.
12. Type of polyp in which invasive carcinoma arose: Not identified.
13. Additional pathologic findings: Not identified
14. Pathological TNM Stage: pT3N0 (According to the eighth edition, American Joint Committee on Cancer Staging Guidelines for Tumors).
15. TNM descriptors: Not applicable.

Gross description:
The specimen consists of a segment of colon, 30 cm in length and 8 cm in proximal cut end circumference, 6 cm in distal circumference, and mesocolic soft tissue. A gray tan firm annular elevated and centrally ulcerative tumor, 8x6 cm, is noted, 3.5 cm to the nearest cut end. On cross section, the tumor invades to the mesocolic soft tissue with 6 cm to the radial margin, and serosal retraction is also noted. The both cut ends are free of tumor grossly. There is a dark red nodule found in the omentum, about 1.2x1.2 cm. Representative part for section: A) proximal cut end B) distal cut end C) vascular cut end D-J) tumor K) omentum nodule L-P) lymph nodes.

Note:
1. The interpretation of MMR panel and recommended studies are as below:
(1) Loss of nuclear expression of MLH1 and PMS2: testing for methylation of the MLH1 promoter and/or mutation of BRAF is indicated (the presence of a BRAF V600E mutation and/or MLH1 methylation suggests that the tumor is sporadic and germline evaluation is probably not indicated; absence of both MLH1 methylation and of BRAF V600E mutation suggests the possibility of Lynch syndrome, and sequencing and/or large deletion/duplication testing of germline MLH1 may be indicated).
(2) Please note: There are exceptions to the above IHC interpretations. These results should not be considered in isolation, and clinical correlation with genetic counseling is recommended to assess the need for germline testing.
2. This case has been peer reviewed by two doctors.
#T-59441_2 #M-81403_2 3 1105 S0000F
#T-C4560_2 #M-09183_2 0 1105 000000
#T-D080B_2 #D4-C1003_2 0 1105 000000
